# Coexistence of Metabolic Neuropathy and Cervical Myelopathy

**DOI:** 10.7759/cureus.114294

**Published:** 2026-08-10

**Authors:** Jasmin K Bhullar

**Affiliations:** 1 Department of Internal Medicine, University of North Carolina Health Southeastern, Lumberton, USA

**Keywords:** cervical myelopathy, diabetes, metabolic syndrome, polyneuropathy, prediabetes

## Abstract

A 63-year-old man with extensive multilevel degenerative cervical and thoracic spine disease, class 1 obesity, essential hypertension, mixed hyperlipidemia, and a history of type 2 diabetes mellitus presented with progressive bilateral upper and lower extremity paresthesias, burning pain in the hands and feet, gait instability, and a history of a fall with transient loss of consciousness. Initial workup revealed a hemoglobin A1c (HbA1c) of 5.4%, and an extensive neuropathy panel was unrevealing. Imaging demonstrated ossification of the posterior longitudinal ligament (OPLL) with multilevel central canal stenosis and cord deformity without cord signal change. The clinical picture was attributed primarily to cervical myelopathy, and the patient underwent anterior cervical discectomy and fusion (ACDF) at C3-C4 and C6-C7. Postoperatively, symptoms failed to improve and progressively worsened, with new-onset dense numbness involving the face and oral mucosa. Serial HbA1c measurements revealed a dramatic rise from 5.4% at initial presentation to 5.9% two months later and ultimately to 13.8% approximately seven months after initial presentation, unmasking poorly controlled type 2 diabetes as a likely major contributor to the patient's length-dependent sensorimotor polyneuropathy. This case underscores that diabetic peripheral neuropathy (DPN) can present and progress even when HbA1c is within the non-diabetic range, particularly in patients with metabolic syndrome, and that structural spinal pathology may coexist with, but not fully explain, progressive neurological symptoms.

## Introduction

Degenerative cervical myelopathy (DCM) and diabetic peripheral neuropathy (DPN) are among the most common neurological conditions encountered in clinical practice, and their coexistence in older adults with metabolic comorbidities presents a significant diagnostic challenge [1,2]. Both conditions can produce bilateral extremity paresthesias, sensory loss, gait instability, and weakness, making clinical differentiation difficult without careful electrodiagnostic and longitudinal assessment [3,4].

A growing body of evidence demonstrates that peripheral nerve damage can begin during the prediabetic phase, well before hemoglobin A1c (HbA1c) reaches the diagnostic threshold for diabetes [5-8]. Metabolic syndrome, comprising obesity, hypertension, dyslipidemia, and impaired glucose metabolism, has been identified as a crucial independent risk factor for polyneuropathy, even in the absence of overt hyperglycemia [9]. Furthermore, diabetes is a well-established predictor of inferior surgical outcomes in cervical spondylotic myelopathy, with diabetic patients demonstrating poorer recovery of sensory function, gait, and bladder function after decompressive surgery [10].

This case report describes a patient in whom progressive length-dependent polyneuropathy was initially attributed to cervical myelopathy from ossification of the posterior longitudinal ligament (OPLL) and multilevel stenosis. Despite surgical decompression, symptoms worsened, and serial glycemic monitoring ultimately revealed rapidly deteriorating glucose control, suggesting that metabolic neuropathy was a major, and potentially the predominant, driver of the patient's neurological decline.

## Case presentation

A 63-year-old man presented to the emergency department after an unwitnessed fall with transient loss of consciousness. He reported losing his balance while walking in his living room, falling, and subsequently awakening seated in a chair with no recollection of how he got there. He endorsed a mild headache but denied new focal weakness, speech difficulty, or bowel/bladder incontinence.

His medical history was significant for type 2 diabetes mellitus (previously on metformin, discontinued approximately two years prior when HbA1c was 5.3%), class 1 obesity, essential hypertension, mixed hyperlipidemia, stage 2 chronic kidney disease, bilateral knee arthritis, diastasis recti, umbilical hernia (repaired approximately five years prior), and extensive degenerative spine disease including multilevel cervical and thoracic spondylosis, cervical and lumbosacral radiculopathy, and a remote history of anterior cervical discectomy and fusion (ACDF) at C5-C6 approximately 20 years prior.

On presentation, the patient reported progressive, severe burning pain and reduced sensation in the palms of his hands and soles of his feet, which had been present for six years and worsening over the past year. He described the sensation of walking on a "thick sponge" under both feet, causing frequent loss of balance. He also reported shooting pain radiating up his arms and neck. These symptoms had been progressively worsening over recent months. He had previously been managed with methadone, clonazepam, and gabapentin through a pain clinic but had discontinued these medications three months prior, seeking definitive treatment rather than chronic pain management.

Vital signs and initial laboratory data

Vital signs on presentation were notable for a blood pressure of 155/67 mmHg, pulse of 56 beats per minute (bpm), respiratory rate of 17 breaths per minute, temperature of 98.6°F, and oxygen saturation of 96% on room air. Basic laboratory studies showed no leukocytosis, kidney function at baseline, and a hemoglobin A1c of 5.4% (Table 1).

**Table 1:** Laboratory test results at the time of admission.

Laboratory test	Result	Reference range
White blood cell count (WBC)	8.0 × 10⁹/L	4.8-10.8 × 10⁹/L
Creatinine	1.16 mg/dL	0.50-1.40 mg/dL
Estimated glomerular filtration rate (eGFR)	71 mL/minute/1.73 m²	≥60 mL/minute/1.73 m²
Hemoglobin A1c (HbA1c)	5.4%	4.7%-5.6%

Imaging

**Figure 1:**
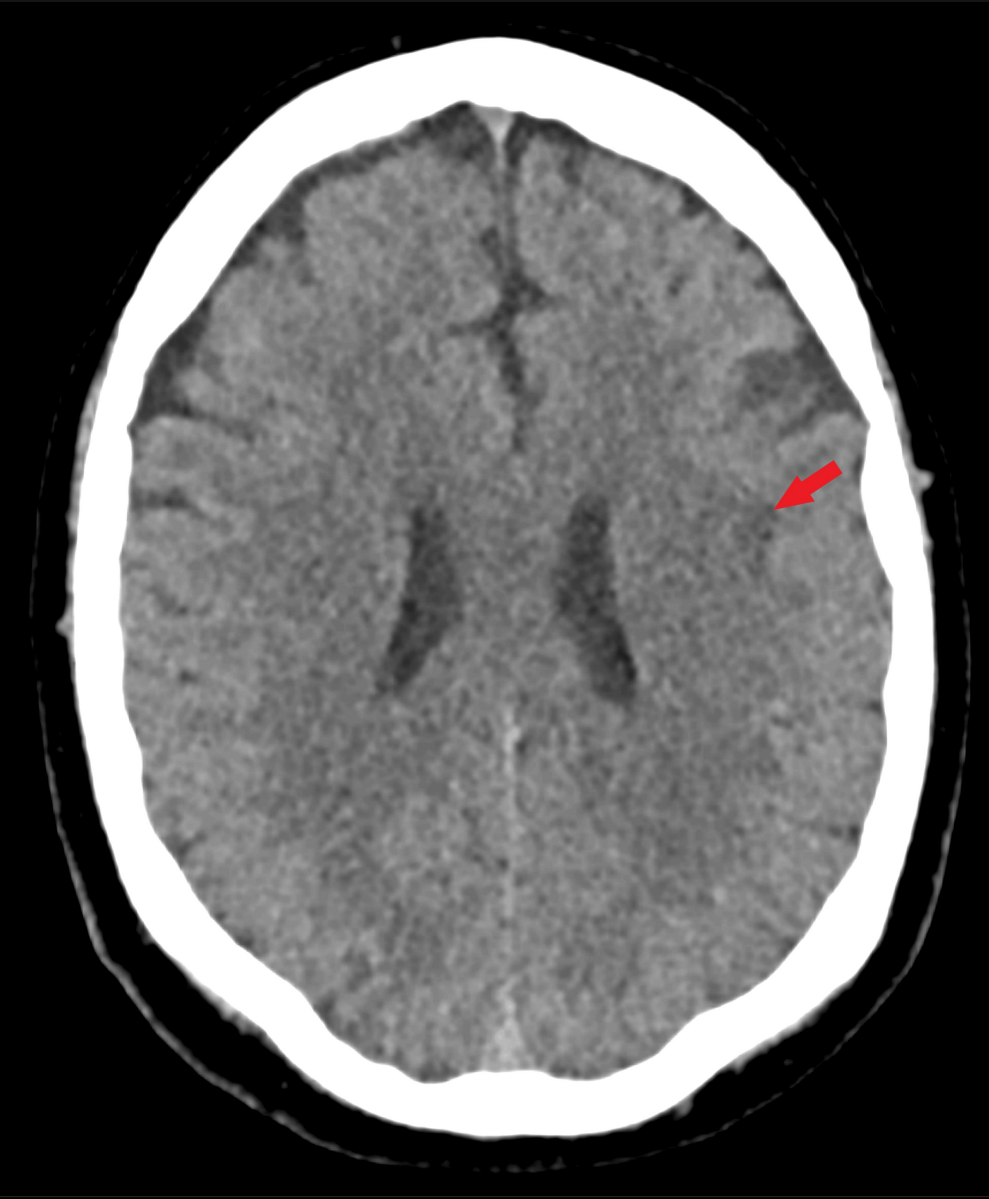
Axial head computed tomography (CT) imaging without contrast demonstrates a hypodensity in the subcortical white matter in the posterior aspect of the left frontal lobe (red arrow).

During this hospitalization, extensive imaging studies were performed, including computed tomography (CT) of the head, magnetic resonance imaging (MRI) of the brain, CT of the spine, and MRI of the spine: CT of the head without contrast revealed an indeterminate 0.6 cm hypodensity in the subcortical white matter of the left frontal lobe posteriorly near the vertex (Figure 1).

**Figure 2:**
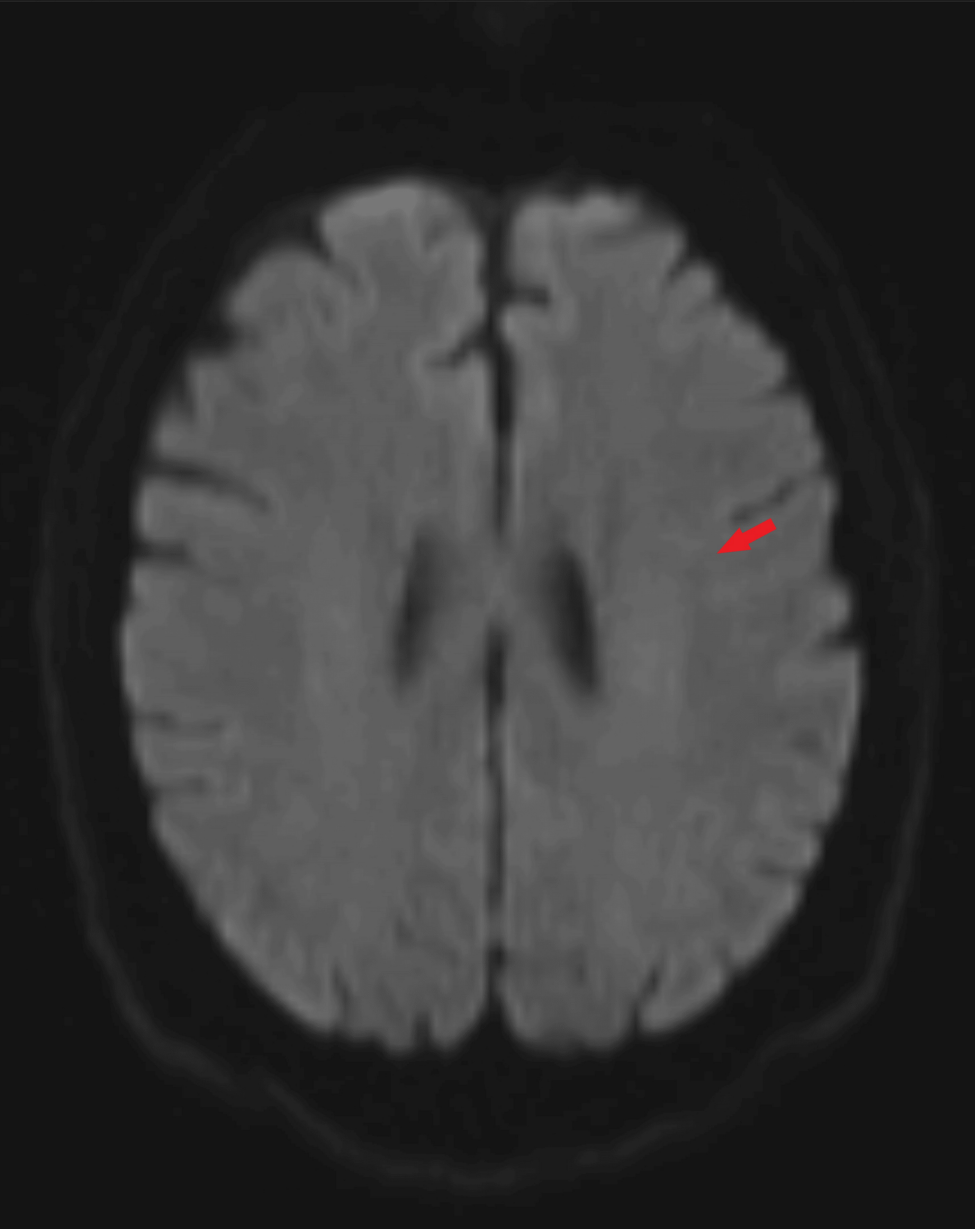
Diffusion-weighted imaging (DWI) axial brain magnetic resonance imaging (MRI) shows no evidence of acute intracranial abnormality in the posterior aspect of the left frontal lobe as the region of interest. This region of interest is marked with a red arrow.

However, an MRI of the brain with and without contrast showed no evidence of acute intracranial abnormality, effectively ruling out acute stroke (Figure 2).

**Figure 3:**
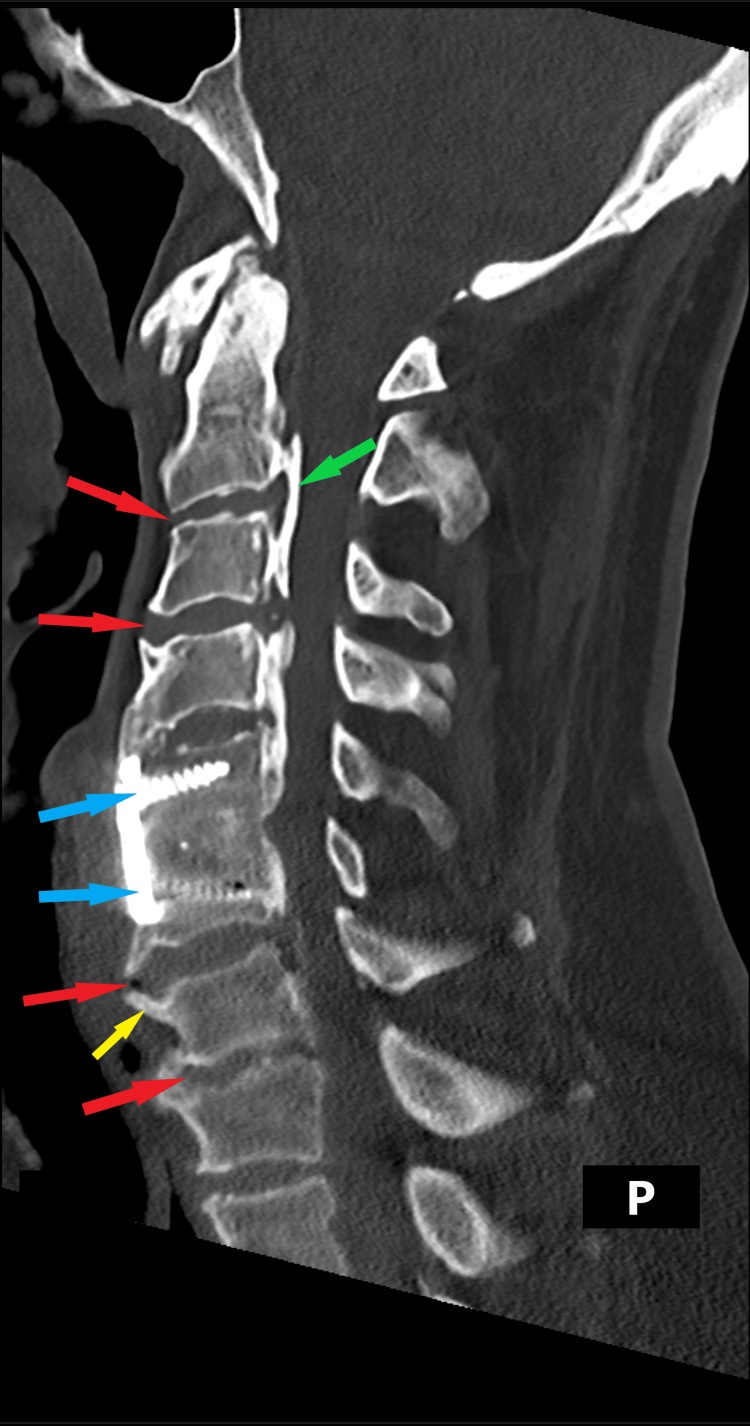
Sagittal computed tomography (CT) of the cervical spine without contrast shows postsurgical change from a prior anterior cervical discectomy and fusion (ACDF) procedure at the C5-C6 level with vertebral screws (blue arrows). It demonstrates multilevel disc space narrowing at the C2-C3, C3-C4, C6-C7, and C7-T1 levels (red arrows). Ossification of the posterior longitudinal ligament is present (green arrow). Degenerative spurring is noted (yellow arrow).

Spine imaging was performed in the setting of persistent neurological deficits. CT of the cervical spine without contrast demonstrated postsurgical, spondylotic, and degenerative changes with multilevel disc space narrowing, degenerative spurring, and ossification of the posterior longitudinal ligament contributing to central canal stenosis, with no acute fracture identified (Figure 3).

**Figure 4:**
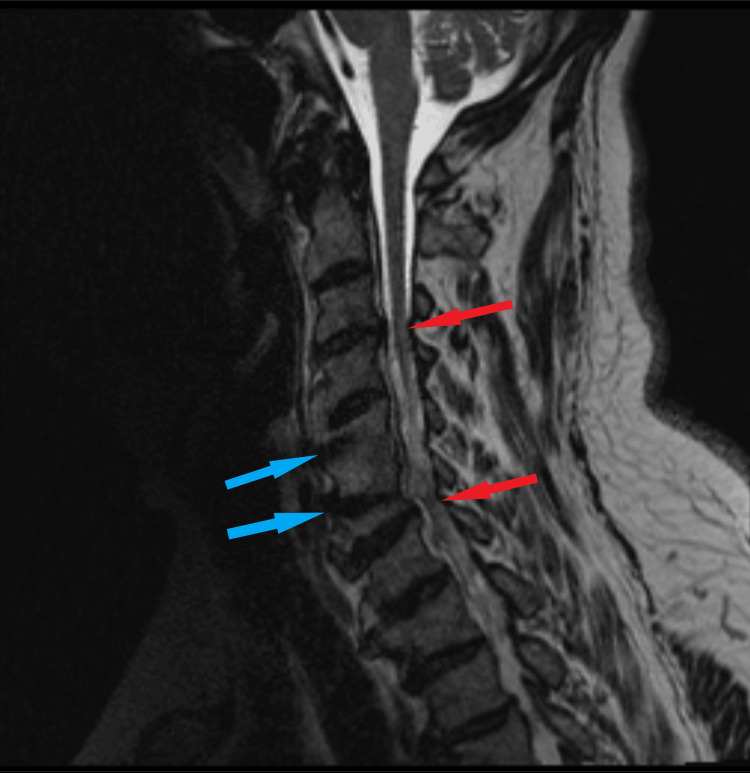
T2-weighted axial magnetic resonance imaging (MRI) of the cervical spine without contrast demonstrates multilevel chronic degenerative changes with central canal stenosis at the C3-C4 and C6-C7 levels (red arrows). Postsurgical changes from a prior anterior cervical discectomy and fusion (ACDF) procedure at the C5-C6 level with fixation hardware including vertebral screws (blue arrows) are present.

MRI of the cervical spine demonstrated multilevel chronic degenerative changes with central canal stenosis and cord deformity, most notably severe stenosis at C6-C7, along with previous C5-C6 ACDF changes; no acute cord edema or myelomalacia was identified (Figure 4).

Neurology was consulted in the emergency department. Physical examination revealed bilateral progressively worsening lower extremity weakness, bilateral upper and lower extremity stocking-glove distribution paresthesias, patchy sensory loss in the extremities, increased tone more pronounced in the bilateral lower extremities, hyperreflexia more pronounced in the bilateral lower extremities, and generalized weakness sparing the face. The combination of upper motor neuron signs (hyperreflexia and increased tone) with length-dependent sensory loss raised concern for both cervical myelopathy and a superimposed peripheral polyneuropathy.

Neurology consultation and hospital course

The neurology team noted the diagnostic dilemma: while the patient had significant structural cervical spine disease, the clinical pattern was also consistent with a length-dependent generalized sensorimotor neuropathy. The consulting neurologist stated that the most common cause of such neuropathies is diabetes, but the patient did not appear diabetic on current workup (HbA1c of 5.4%), and B12 deficiency had been excluded. It was acknowledged that once common etiologies are excluded, the differential for polyneuropathy encompasses numerous potential etiologies and may remain elusive despite aggressive workup. An extensive neuropathy workup was initiated by the neurologists during this hospitalization. Its results are summarized in the table below (Table 2).

**Table 2:** Results of laboratory workup for neurological symptoms. SSA, Sjögren's syndrome A; SSB, Sjögren's syndrome B

Laboratory test	Result	Reference range
Vitamin B12	829 pg/mL	226-966 pg/mL
Folate	11.0 ng/mL	3.0-17.5 ng/mL
Vitamin B1 (thiamine)	129 nmol/L	70-180 nmol/L
Vitamin B6 (pyridoxine)	18 mcg/L	5-50 mcg/L
Vitamin E	5.6 mg/L	5.5-17.0 mg/L
Rheumatoid factor (RF)	<3.5 IU/mL	<14.0 IU/mL
Anti-SSA antibody	Negative	Negative
Anti-SSB antibody	Negative	Negative
Serum protein electrophoresis (SPEP)	No monoclonal protein detected	No monoclonal protein
Immunoglobulin G anti-GM1 (IgG anti-GM1)	Negative	Negative
Immunoglobulin M anti-GM1 (IgM anti-GM1)	Negative	Negative
Immunoglobulin M anti-disialoganglioside GD1b (IgM anti-GD1b)	Negative	Negative
Anti-GQ1b immunoglobulin G (GQ1b-IgG) enzyme-linked immunosorbent assay (ELISA)	Negative	Negative
Glutamic acid decarboxylase 65 antibody (GAD65 Ab)	0.00 nmol/L	≤0.02 nmol/L

After having obtained the results of the MRI, the progressive gait dysfunction and sensory loss were felt to be more consistent with acute-on-chronic worsening of cervical myelopathy.

The neurology team emphasized that spondylotic myelopathy tends to have a subacute presentation that can acutely worsen with minor trauma such as falls, fitting the patient's clinical trajectory. They noted that the patient was still ambulatory but required a walker and recommended urgent neurosurgical evaluation to prevent further neurological injury, given the risk of quadriplegia or quadriparesis with another head or neck injury. Despite the absence of cord signal change on MRI, the neurologist emphasized that patients can be symptomatic from severe stenosis even without this finding.

The patient was admitted for observation and workup. Multiple attempts to arrange neurosurgical transfer were declined by receiving facilities. The general impression of the neurosurgeons was that the findings on the imaging studies were chronic and that the patient did not need any emergent surgical intervention. The patient was discharged with outpatient referrals to neurology and neurosurgery.

Surgical intervention

Approximately three months after initial presentation, the patient underwent ACDF at C3-C4 and C6-C7 with standalone cages.

Postoperative course

Approximately two weeks after discharge from the surgical hospitalization, the patient presented to the emergency department with worsening bilateral lower extremity weakness, numbness, and lower back pain. He denied saddle anesthesia, urinary or fecal incontinence, or history of cancer. Examination revealed 4/5 strength in bilateral lower extremities with diminished sensation. He was able to ambulate with a steady gait but reported subjective difficulty. He was discharged with a short course of oral steroids (prednisone 20 mg daily for 14 days), pregabalin, a lidocaine patch, and a one-time dexamethasone injection, with a neurosurgery referral.

Approximately three months postoperatively, the patient presented with continued and worsening symptoms, now including new dense numbness involving the top of his head, face, and mouth, along with burning in his mouth affecting his ability to eat solid food. He reported severe difficulty rising from a chair due to profound weakness and extreme difficulty walking due to dense numbness in his feet. Pain patches prescribed as an outpatient had been ineffective. Laboratory evaluation two months later revealed a markedly elevated HbA1c of 13.8%. The trajectory of his HbA1c values is shown in the table below (Table 3). The patient's hyperglycemia had previously been managed with metformin, which was discontinued 905 days prior to the initial presentation after achieving an HbA1c of 5.3%. He subsequently presented to the hospital 203 days after the initial evaluation and was found to be in diabetic ketoacidosis.

**Table 3:** Serial hemoglobin A1c (HbA1c) measurements over time.

Timepoint	Clinical context	HbA1c (%) (reference range: 4.7%-5.6%)
2732 days prior to presentation	At primary care physician office visit	6.3
905 days prior to presentation	At primary care physician office visit	5.3
Initial presentation	Initial presentation after fall	5.4
61 days after initial presentation	At primary care physician office visit	5.9
203 days after initial presentation	During hospitalization for diabetic ketoacidosis	13.8

The patient had not been on any antidiabetic medications for approximately two years following metformin discontinuation. While he did receive a brief course of corticosteroids postoperatively (a one-time dexamethasone injection and a short oral steroid course), this would not account for the magnitude or trajectory of HbA1c elevation observed.

## Discussion

This case illustrates several important clinical principles regarding the intersection of degenerative spine disease and metabolic neuropathy.

Diabetic neuropathy can precede overt hyperglycemia

The most striking feature of this case is the patient's progressive sensorimotor polyneuropathy in the setting of an initially non-diabetic HbA1c. A substantial body of evidence supports the concept that peripheral nerve damage can begin during the prediabetic phase. In the PROMISE cohort, the prevalence of peripheral neuropathy was 49% in individuals with prediabetes compared with 50% in those with new-onset diabetes and 29% in normoglycemic individuals [6]. The NHANES survey found peripheral neuropathy in 7.5%-16% of individuals with prediabetes [11]. A Lancet Neurology review concluded that nerve damage might begin long before individuals with hyperglycemia develop overt diabetes, and a growing body of evidence supports an association between prediabetes and small-fiber symptoms [5].

This patient had multiple metabolic syndrome components: class 1 obesity, essential hypertension, mixed hyperlipidemia, and a known history of type 2 diabetes. Metabolic syndrome has been identified as a crucial risk factor for DPN in type 2 diabetes, and the presence of more metabolic syndrome components increases the likelihood of neuropathy [12]. Metabolic syndrome components are independently associated with an increased risk of polyneuropathy regardless of glycemic status [9].

The diagnostic challenge of coexisting myelopathy and polyneuropathy

Differentiating cervical myelopathy from peripheral neuropathy is particularly challenging when both conditions coexist. Coexisting diabetic neuropathy may obscure the classic examination findings of cervical spondylotic myelopathy by attenuating expected hyperreflexia and producing non-dermatomal patterns of extremity numbness [13]. In this patient, the examination demonstrated features of both upper motor neuron dysfunction (hyperreflexia and increased tone in lower extremities) and peripheral neuropathy (stocking-glove sensory loss and burning dysesthesias), creating a mixed clinical picture.

The consulting neurologist recognized this dilemma, noting that while structural cervical spine disease was present, the pattern of symptoms was also consistent with a length-dependent generalized sensorimotor neuropathy. However, the severity of the structural disease, OPLL with multilevel stenosis and cord deformity, appropriately prompted surgical intervention to prevent catastrophic neurological injury.

Persistent symptoms after surgical decompression

The failure of symptoms to improve, and indeed their worsening, after surgical decompression is consistent with the literature on surgical outcomes in diabetic patients with cervical myelopathy. Diabetic patients demonstrate significantly lower recovery rates for lower extremity motor function (36.1% versus 43.4%), upper extremity sensory function (36.8% versus 49.6%), and bladder function compared with non-diabetic patients [10]. Diabetes has been identified as a significant independent risk factor for poor somatosensory conduction recovery after cervical spine surgery (OR, 4.52; 95% CI, 2.32-7.12), and this effect correlates with preoperative HbA1c levels [14]. Furthermore, hyperglycemia may directly impair the postoperative healing of the spinal cord [15].

In this patient, the development of new symptoms not explained by cervical pathology, particularly dense numbness of the scalp, face, and oral mucosa with burning dysesthesias affecting eating, further supports a systemic process such as polyneuropathy rather than a purely structural etiology.

Implications of metformin discontinuation

An important consideration in this case is the discontinuation of metformin approximately two years prior to presentation, when HbA1c was 5.3%. Beyond its glucose-lowering effects, emerging evidence suggests that metformin may have neuroprotective properties in diabetic peripheral neuropathy, with metformin-treated patients demonstrating lower tibial nerve cross-sectional area (indicating less nerve swelling/damage) and reduced neuropathy symptom severity compared with matched non-metformin-treated controls [16]. The cessation of metformin may have contributed to the loss of glycemic control.

Clinical implications

When patients with multilevel degenerative spine disease and metabolic risk factors present with progressive polyneuropathy symptoms, clinicians should maintain a broad differential and avoid attributing all symptoms to structural pathology alone. The presence of a compressive lesion on imaging does not exclude a concurrent systemic process affecting peripheral nerves.

This case highlights that suspected metabolic neuropathy may coexist with cervical myelopathy, even in patients undergoing treatment for cervical myelopathy. Serial glycemic monitoring may help identify changes in glycemic status over time, even when the initial HbA1c is not in the diabetic range. Dysglycemia may have contributed to the patient's neurological symptoms. However, other potential contributors, including corticosteroid exposure and alternative metabolic etiologies, should also be considered. The patient's craniofacial sensory symptoms were atypical for diabetic neuropathy. Further diagnostic evaluation, including electromyography and nerve conduction studies, neither of which was performed in this patient, may be helpful in further characterizing the neuropathy.

Persistent or worsening neurological symptoms after adequate surgical decompression should prompt reassessment for alternative or coexisting etiologies, including metabolic neuropathy. A simultaneous development or progression of a systemic process affecting peripheral nerves may mimic, overlap with, or amplify symptoms that were initially attributed to compression. Failure to identify these coexisting diseases can lead to unnecessary revision surgery and the delayed treatment of the underlying neurological cause.

The discontinuation of metformin in patients with a history of type 2 diabetes warrants close glycemic surveillance, as rebound hyperglycemia may develop insidiously and contribute to neuropathy progression. In this case, the cessation of metformin likely allowed glycemic deterioration, underscoring the importance of continued metabolic monitoring even when diabetes appears to be in remission.

## Conclusions

This case demonstrates that diabetic peripheral neuropathy can present and progress even when HbA1c is within the non-diabetic range, particularly in patients with metabolic syndrome. The coexistence of severe multilevel degenerative cervical spine disease created a diagnostic attribution bias toward structural myelopathy, and the patient's symptoms persisted despite surgical decompression. The subsequent dramatic rise in the HbA1c over approximately seven months unmasked poorly controlled type 2 diabetes as a likely major contributor to the progressive polyneuropathy.

This case underscores the importance of maintaining a broad differential diagnosis, performing serial glycemic monitoring, and recognizing that metabolic neuropathy may coexist with and mimic structural myelopathy, particularly in the growing population of patients with obesity and metabolic syndrome.
